# Spatial and temporal village-level prevalence of *Plasmodium* infection and associated risk factors in two districts of Meghalaya, India

**DOI:** 10.1186/s12936-021-03600-w

**Published:** 2021-02-04

**Authors:** Anne Kessler, Badondor Shylla, Upasana Shyamsunder Singh, Rilynti Lyngdoh, Bandapkupar Mawkhlieng, Anna Maria van Eijk, Steven A. Sullivan, Aparup Das, Catherine Walton, Mark L. Wilson, Jane M. Carlton, Sandra Albert

**Affiliations:** 1 Center for Genomics and Systems Biology, Department of Biology, New York University, New York, NY 10003 USA; 2 Indian Institute of Public Health-Shillong, Shillong, Meghalaya 793001 India; 3 Martin Luther Christian University, Shillong, Meghalaya 793006 India; 4 Department of Earth and Environmental Sciences, School of Natural Sciences, University of Manchester, Manchester, M13 9PT UK; 5 Department of Health Services (Malaria), National Vector Borne Disease Programme, Lawmali, Pasteur Hill, Shillong, Meghalaya 793001 India; 6 ICMR-National Institute of Research in Tribal Health, Jabalpur, Madhya Pradesh 482003 India; 7 Department of Epidemiology, School of Public Health, University of Michigan, Ann Arbor, MI 48109 USA; 8 Department of Epidemiology, College of Global Public Health, New York University, New York, NY 10012 USA

**Keywords:** Subpatent *Plasmodium* infections, *Anopheles* mosquito abundance, Declining incidence, Malaria elimination

## Abstract

**Background:**

Despite declining incidence over the past decade, malaria remains an important health burden in India. This study aimed to assess the village-level temporal patterns of *Plasmodium* infection in two districts of the north-eastern state of Meghalaya and evaluate risk factors that might explain these patterns.

**Methods:**

Primary Health Centre passive malaria case data from 2014 to 2018 were analysed to characterize village-specific annual incidence and temporal trends. Active malaria case detection was undertaken in 2018 and 2019 to detect *Plasmodium* infections using PCR. A questionnaire collected socio-demographic, environmental, and behavioural data, and households were spatially mapped via GPS. Adult mosquitoes were sampled at a subset of subjects' houses, and *Anopheles* were identified by PCR and sequencing. Risk factors for *Plasmodium* infection were evaluated using bivariate and multivariate logistic regression analysis, and spatial cluster analysis was undertaken.

**Results:**

The annual malaria incidence from PHC-based passive surveillance datasets in 2014–2018 was heterogenous but declining across villages in both districts. Active surveillance in 2018 enrolled 1468 individuals from 468 households (West Jaintia Hills) and 1274 individuals from 359 households (West Khasi Hills). *Plasmodium falciparum* prevalence per 100 people varied from 0 to 4.1% in the nine villages of West Jaintia Hills, and from 0 to 10.6% in the 12 villages of West Khasi Hills**.** Significant clustering of *P. falciparum* infections [observed = 11, expected = 2.15, Relative Risk (RR) = 12.65; *p* < 0.001] was observed in West Khasi Hills. A total of 13 *Anopheles* species were found at 53 houses in five villages, with *Anopheles jeyporiensis* being the most abundant. Risk of infection increased with presence of mosquitoes and electricity in the households [Odds Ratio (OR) = 1.19 and 1.11], respectively. Households with reported animals had reduced infection risk (OR = 0.91).

**Conclusion:**

Malaria incidence during 2014–2018 declined in all study villages covered by the passive surveillance data, a period that includes the first widespread insecticide-treated net campaign. The survey data from 2018 revealed a significant association between *Plasmodium* infection and certain household characteristics. Since species of *Plasmodium*-competent mosquito vectors continue to be abundant, malaria resurgence remains a threat, and control efforts should continue.

## Background

In 2018, India (4%) was one of five countries that accounted for close to 50% of all malaria cases worldwide, along with Nigeria (25%), Democratic Republic of the Congo (11%), Mozambique (5%), and Uganda (4%) [1]. The regions of India with high malaria incidence have more recently been found in the east and northeast of the country, with six states contributing roughly three-quarters of cases [2]. In the seven northeastern states, malaria is generally declining [3], but continues to impede the equitable health improvement and socioeconomic development of the region. Historically, the state of Meghalaya has reported more than 20% of cases annually in this region [2]. Among the Meghalaya districts endemic for malaria, the majority of recent cases have been observed in the Garo Hills, West Khasi Hills, and West Jaintia Hills [4]. Malaria cases and fatalities increased in Meghalaya from 2012 to 2015, but declined in 2016 [4]. While anti-malarial drug therapy could be responsible for the decline, the causality is not clear, as artemether-lumefantrine (AL) against *Plasmodium falciparum* was introduced in the northeastern states of India in 2013 and has not changed since then [2, 3]. Alternately, the decline could be related to the > 941,000 long-lasting insecticidal nets (LLINs) distributed throughout the state for the first time in mid-2016 by the Meghalaya state malaria control programme.

Other than unpublished findings from Meghalaya government reports, no data on recent changes in incidence are available, nor on risk factors associated with malaria in this region. This study assessed the village-level prevalence of *Plasmodium* infection, explored geo-spatial patterns, and evaluated village-level risk factors in two districts of Meghalaya.

## Methods

### Study setting and ethical approval

**Fig. 1:**
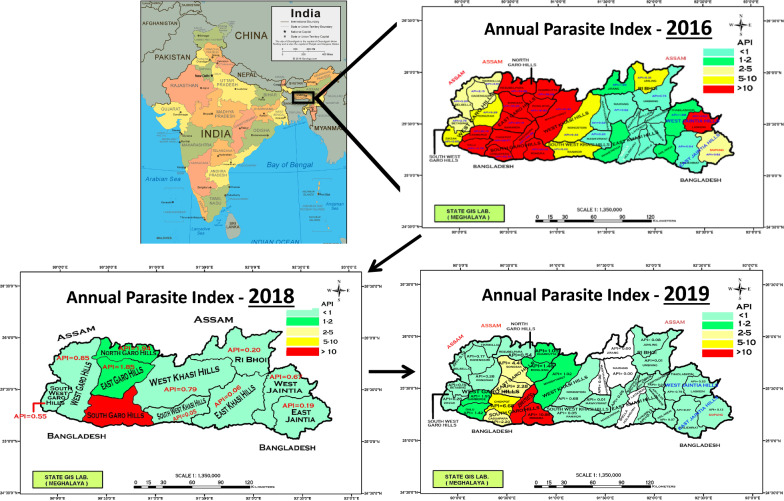
Malaria Annual Parasite Index (API) map of Meghalaya state in northeastern India by district for 2016, 2018 and 2019

Data were gathered from two districts of Meghalaya state: West Khasi Hills and West Jaintia Hills. The districts were selected based on the 2016 Annual Parasite Index (API) reported for all 11 Districts (Fig. 1). Although the API was highest in the Garo Hills at this time, these districts were not selected for active case detection due to logistical issues and safety concerns in the surrounding area(s). Two types of malaria data were analysed: passive case data obtained from the district Primary Health Centres (PHC) during 2014–2018 and active case surveillance during 2018 and 2019.

Ethical approval for the study was obtained from the Institutional Review Boards (IRBs) of Martin Luther Christian University, Shillong, Meghalaya, India and New York University, New York, NY, USA. Written informed consent was obtained from all the participants who were 18 years of age or older. Assent was obtained for the participants aged 7–17 years in addition to parental consent.

### Data sources and management for passive case detection

Malaria case data were collected from laboratory logbooks and Accredited Social Health Activists (ASHA) reports for January 2014 to December 2018 at the Barato PHC (West Jaintia Hills), and January 2015 to December 2018 at the Nonglang PHC (West Khasi Hills). Case data for Kyrdum, a West Khasi Hills village in the Nonglang PHC catchment area surveyed here by active surveillance, was not available and, therefore, not included in the dataset. These datasets include village-level malaria surveillance details on the number of blood samples tested and the number that were positive for either *Plasmodium* and/or *P. falciparum* infection. Laboratory-confirmed *Plasmodium* infections were those with parasites in the peripheral blood smear as detected through microscopy or through an antigen-detecting Rapid Diagnostic Test (RDT) (Source: NVBDCP guidelines 2013).

### Sampling design for active case surveillance

Active case surveillance was conducted in a subset of West Jaintia Hills and West Khasi Hills villages in 2018 and 2019 using a community-based survey. Similar to the district selection, the Barato PHC from West Jaintia Hills and the Nonglang PHC from West Khasi Hills were chosen for sampling based on API relative to all other PHCs within the respective districts. Nine of the 20 villages under the Barato PHC (West Jaintia Hills), and 12 of 32 villages under the Nonglang PHC (West Khasi Hills) were selected for surveying based on API, logistics, and representation across both the PHCs and their regional sub-centres. To achieve the calculated study sample size, 50% of households in each village were selected using the Probability Proportion to Size (PPS) technique at random from the sampling frame provided by the ASHA in each village. Additional households were sampled on a need basis to fulfill target enrollment numbers. All eligible consenting individuals from each selected household were enrolled; inclusion criteria were defined as individuals between the ages of 1 and 69 years with no immediate health risks and apparent full comprehension of the study procedures. In the event that not all household members were present at the time of initial enrollment, a repeat visit was made on the same day to capture all eligible participants.

### Survey data collected from community survey

Data were collected from April to November 2018 and May to September 2019 in both study regions, i.e., West Khasi Hills and West Jaintia Hills (Table 1). All households with enrolled study participants were spatially mapped via Global Positioning System (GPS) readings. Answers to a household survey were given by one resident, most commonly the ‘head of household,’ followed by individual surveys administered in the local language to each individual resident of the household, consisting of a pre-tested, structured interview with mostly close-ended questions. Demographic information (age, gender, education, occupation), medical history (fever, episodes of malaria, antimalarial use in the past year, other complaints), travel history over the past two weeks, and data on use of malaria prevention methods (e.g., insecticide-treated nets (ITNs), repellents, coils) were also obtained. Daily behaviours and practices were also surveyed, with a focus on those that may put individuals at risk of infection, such as participation in outdoor activities during the evening. Parents or caretakers responded to some questions on behalf of their child(ren) for questions where the child did not have the knowledge or understanding to provide an answer.

**Table 1:** Population of West Jaintia Hills and West Khasi Hills villages and the number of households and residents enrolled in the study during 2018 and 2019

District	Villages	Total Population	Total Households	Households enrolled (2018) n (%)	Individuals enrolled (2018) n (%)	Households enrolled (2019) n (%)	Individuals enrolled (2019) n (%)	New Enrollees (2019)
West Jaintia Hills	Barato A	597	155	107 (69.03)	389 (65.16)	42 (27.10)	189 (31.66)	70
	Barato B	652	128	62 (48.44)	171 (26.23)	43 (33.59)	194 (29.75)	110
	Barato C	465	99	65 (65.66)	196 (42.15)	40 (40.40)	173 (37.20)	81
	Barato E	569	100	53 (53.00)	110 (19.33)	0	0	0
	Iongkwang	420	83	32 (38.55)	116 (27.62)	0	0	0
	Kynshur	643	125	43 (34.40)	114 (17.73)	0	0	0
	Mukroh A	500	100	60 (60.00)	178 (35.60)	0	0	0
	Mukroh B	439	65	34 (52.31)	106 (24.15)	0	0	0
	Saba	587	85	30 (35.29)	88 (14.99)	0	0	0
Total		4872	940	468 (49.79)	1468 (30.13)	117 (12.45)	556 (11.41)	261
West Khasi Hills	Khyllemsangrin	413	68	29 (42.65)	111 (26.88)	9 (13.24)	33 (7.99)	4
	Kriangrin	300	68	34 (50.00)	105 (35.00)	15 (22.06)	42 (14.00)	6
	Kyndongnei	187	32	25 (78.13)	133 (71.12)	0	0	0
	Kyrdum	621	93	50 (53.76)	165 (26.57)	13 (13.98)	48 (7.73)	15
	Langja	723	122	16 (13.11)	20 (2.77)	0	0	0
	Mawlan B	109	21	11 (52.38)	34 (31.19)	6 (28.57)	6 (5.50)	2
	Mawsikar	468	72	28 (38.89)	99 (21.15)	4 (5.56)	14 (2.99)	2
	Nonglang	365	73	56 (76.71)	209 (57.26)	24 (32.88)	65 (17.81)	13
	Pyndengsohstap	47	10	5 (50.00)	19 (40.43)	2 (20.00)	7 (14.89)	1
	Siangra	251	36	22 (61.11)	66 (26.29)	10 (27.78)	12 (4.78)	0
	Umthlu	398	68	33 (48.53)	120 (30.15)	20 (29.41)	44 (14.00)	4
	Umwahlang	552	85	50 (58.82)	193 (34.96)	2 (2.35)	7 (1.27)	0
Total		4434	748	359 (47.99)	1274 (28.73)	105 (14.04)	278 (6.27)	47

### Blood sample collection and processing

Blood was taken by finger prick with a disposable lancet. A bivalent RDT (FalciVax) and an ultra-sensitive RDT (Abbott Alere) were used for point-of-care detection of *P. falciparum* and/or *P. vivax* and *P. falciparum* infections, respectively. A small blood volume was taken into a microvette, and post-centrifugation the blood components were separated and stored at -80 C°. DNA was extracted from the RBCs. Additionally, blood was spotted on to Whatman filter paper and smeared onto glass slides. The thin blood smears were fixed in methanol and dried prior to Giemsa staining and subsequent qualitative and quantitative evaluation by light microscopy. Laboratory-confirmed *Plasmodium* infections were those with parasites in the peripheral blood as detected via RDT and/or Polymerase Chain Reaction (PCR) only (no infections were detected by microscopy).

### Species-specific PCR detection of *Plasmodium* parasites

*Plasmodium falciparum* and/or *P. vivax* infections were detected in the community survey study participants by PCR amplification of concentrated DNA extracted from microvettes. Briefly, DNA was extracted using the QIAamp DNA mini kit (QIAGEN) and eluted in 50 ul of distilled water. The total volume of DNA for each sample was concentrated using a Speed-Vac (Thermo Scientific) to 20% of the starting volume. Two novel genetic markers, Pvr47 present as 14 copies in the *P. vivax* genome, and Pfr364 present as 41 copies in the *P. falciparum* genome, were utilized in a single-step PCR as described [5] using 5 µl of concentrated DNA. PCR amplicons were visualized by standard gel electrophoresis of the entire PCR reaction using ethidium bromide and UV light documentation.

### Mosquito capture methods

Adult mosquitoes were collected using CDC light traps (John W. Hock, Gainesville, FL, USA) during August through November 2018 from two villages in West Khasi Hills (30 households) and three villages in West Jaintia Hills (23 households). One unbaited trap per night per household was hung to the roof or ceiling outside of the room where people sleep to capture human host-seeking mosquitoes. The collected mosquitoes were morphologically identified at the genus level under a microscope in the field, and all *Anopheles* spp. were stored individually in beam capsules and desiccated by storage with silica gel for subsequent species identification by PCR (and sequencing).

### PCR-based identification of *Anopheles* species

DNA extracted from 161 individual adult mosquito specimens (QIAamp DNA Mini kit) was amplified using either the Internal Transcribed Spacer 2 (ITS2) region of ribosomal DNA (101 specimens) or a region of the mitochondrial cytochrome oxidase subunit I (COI) gene (60 specimens). The ITS2 primers were forward primer 5.8S (5′-TGTGAACTGCAGGACACATG-3′) and reverse primer 28S (5′-ATGCTTAAATTTAGGGGGTA-3′) [6, 7]. Each 25 ul reaction consisted of 1.5 µl DNA template and 0.25 µl Phusion High-Fidelity DNA Polymerase with final concentrations of 1X HF Phusion buffer, 200 µM [dNTPs], and 0.5 μM primers. PCR conditions were: 98 °C for 30 s followed by 35 cycles of 98 °C for 10 s, 61 °C for 30 s, and 72 °C for 30 s, with a final extension at 72 °C for 5 min. PCR products were purified using GeneElute™ PCR Clean-up kit (Sigma-Aldrich). Sanger sequencing was performed in the forward direction using the 5.8S forward primer. The COI fragment was amplified using primers LCO-1490 (5′-GGTCAACAAATCATAAAGATATTGG-3′) and HCO-2198 (5′-TAAACTTCAGGGTGACCAAAAATCA-3′) [8]. Each 25 ul reaction consisted of 1 µl of DNA template and 0.15 µl of *Invitrogen* (5 U/µl) Taq DNA Polymerase with final concentrations of 1X (Thermo Scientific™) buffer, 200 µM [dNTPs] (Thermo Scientific™), each primer at 0.16 μM, 2 mM [MgCl_2_], and 0.2 mg/ml [BSA]. The PCR conditions were 95 °C for 2 min followed by 35 cycles of 95 °C for 45 s, 55 °C for 1 min, and 72 °C for 1 min, with a final extension at 72 °C for 7 min. The PCR products were purified using EXOSAP, and Sanger sequencing was subsequently performed in the reverse direction using the HCO-2198 reverse primer.

Sequences were edited as necessary for accurate base calling, and primer sequences were removed using Geneious Prime® 2020.1.2 (http://www.geneious.com) [9]. ITS2 sequences were compared against the NCBI nucleotide database using BLAST. For COI, sequences were compared against the BOLD database (http://www.boldsystems.org) [10] to determine *Anopheles* species identity. If the query search produced two species matches, the ‘phylogenetic tree’ generated by BOLD using public and private COI sequences was referred to in order to confirm the closest match. The sequences generated in this study were deposited in GenBank with Accession Numbers as follows: *Anopheles jeyporiensis*: MT863705 (COI), MT872791 (ITS2); *Anopheles maculatus*: MT863706 (COI), MT862759 (ITS2); *Anopheles pseudowillmori*: MT871948 (COI), MT872797 (ITS2); *Anopheles nivipes*: MT863711 (COI), MT872792 (ITS2); *Anopheles philippinensis*: MT872793 (ITS2); *Anopheles barbirostris*: MT863707 (COI)i; *Anopheles dissidens*: MT872790 (ITS2); *Anopheles nitidus*: MT863712 (COI), MT872789 (ITS2); *Anopheles peditaeniatus*: MT872794 (ITS2); *Anopheles xui*: MT871949 (COI); *Anopheles vagus*: MT872798 (ITS2); *Anopheles splendidus*: MT872799 (ITS2); *Anopheles jamesii*: MT871938 (COI).

### Statistical analysis

*Plasmodium* infection prevalence per 100 people was calculated for each village included in the survey. Associations between demographic, environmental, and behavioral risk factors and village-level *Plasmodium* infection prevalence were determined using logistic regression analysis and odds ratios (OR) (95% confidence intervals (CI)). Risk factors with significant associations were analysed using multivariate logistic regression to adjust for confounders. All data were analysed using Statistical Package of Social Sciences (SPSS) version 15.0 (SPSS Inc., Chicago, IL) and Stata version 14.4 (StataCorp, College Station, TX).

### Spatial cluster analysis

SaTScan 9.6 software (https://www.satscan.org/) was used to identify significant spatial clustering of *Plasmodium* infection prevalence among the study villages. The statistical test employs a circular window to systematically search for significant spatial clusters over a defined geographic area. The radius of the window may vary from zero to a user defined upper limit, here defined as the geographic area that included 50% of the study region to allow for the detection of both small and large clusters [11]. Likelihood ratios were calculated, and p-values were derived by conducting Monte-Carlo replications of the dataset using the continuous Poisson model [12]. The number of individuals with *Plasmodium* infection in each village was defined as ‘cases.’ The total number of people sampled per village was defined as the ‘population.’

## Results

### Trends in malaria incidence from Meghalaya state data 2014–2018

**Fig. 2:**
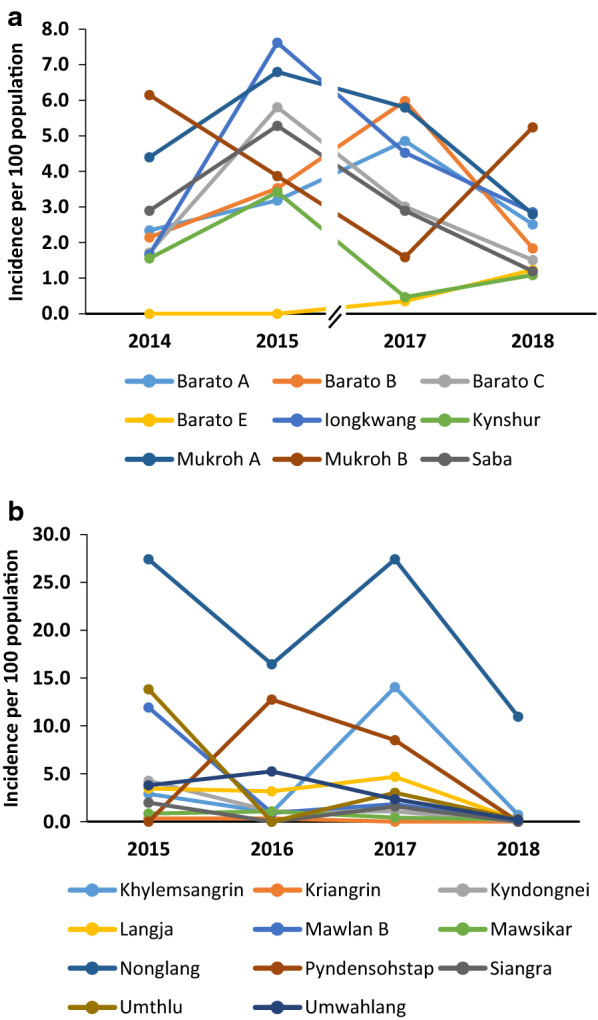
Annual trend of malaria incidence from government health facilities in villages served by **a** Barato PHC, West Jaintia Hills from 2014 to 2018 (2016 not available), and **b** Nonglang PHC, West Khasi Hills from 2015 to 2018

Analysis of government data obtained for West Jaintia Hills and West Khasi Hills indicated temporal patterns in malaria incidence that were similar across villages (Fig. 2). Most of the villages in West Jaintia Hills experienced increasing incidence in malaria cases (primarily *P. falciparum*) between 2014 and 2015 (2016 disaggregated village level data were not available), and declined thereafter, except in Barato A and Barato B, where case numbers continued to increase until 2017 (Fig. 2a).

For villages in West Khasi Hills, malaria incidence either decreased slightly or remained stable between 2015 and 2016 (2014 data were not available), except in Nonglang, Langja, and Khylleimsangrin, where incidence was still increasing until 2017 (Fig. 2b). A decline in incidence was observed for all villages between 2017 and 2018. Nonglang village reported the highest malaria incidence throughout the study period.

### Village-level *Plasmodium* infection prevalence from active surveillance in 2018 and 2019

A a total of 1468 individuals from 468 households were enrolled in the West Jaintia Hills, and 1274 individuals from 359 households were enrolled in the West Khasi Hills (Table 1). The village-level prevalence of *P. falciparum* infection identified through active surveillance in 2018 and as detected by PCR varied from 0 to 4.1% *P. falciparum* (16 PCR positive infections total) in the nine villages of West Jaintia Hills and from 0 to 10.6% *P. falciparum* (17 PCR positive infections total) in the 12 villages of West Khasi Hills (Table 2). No *P. vivax* infections were identified. All 16 *P. falciparum* infections identified in the West Jaintia Hills came from a single village (Barato A), and of the 12 villages in West Khasi Hills, *P. falciparum* infections were identified in six villages, with Siangra showing the highest prevalence (10.61%; Table 2). Of the 33 *P. falciparum* infections identified by PCR in 2018, only the single infection identified in Umwahlang village WKH was also RDT positive. The village-level prevalence of *P. falciparum* infection identified through active surveillance in 2019 was 0% in all villages by all detection methods, i.e., microscopy, RDT, and PCR.

**Table 2.** 2018 village-level prevalence of *Plasmodium* infection

District	Village	No. people enrolled	No. people PCR-pos	Prevalence per 100 population
West Jaintia Hills	Barato A	389	16	4.11 (2.14, 6.08)
	Barato B	171	0	0 (0, 0.02)
	Barato C	196	0	0 (0, 0.02)
	Barato E	110	0	0 (0, 0.03)
	Iongkwang	116	0	0 (0, 0.03)
	Kynshur	114	0	0 (0, 0.03)
	Mukroh A	178	0	0 (0, 0.02)
	Mukroh B	106	0	0 (0, 0.03)
	Saba	88	0	0 (0, 0.04)
Total		1468	16	1.09 (0.56, 1.62)
West Khasi Hills	Khyllemsangrin	111	3	2.7 (0, 5.72)
	Kriangrin	300	1	0.33 (0, 0.98)
	Kyndongnei	133	0	0 (0, 0.02)
	Kyrdum	165	0	0 (0, 0.02)
	Langja	20	0	0 (0, 0.16)
	Mawlan B	34	0	0 (0, 0.10)
	Mawsikar	99	0	0 (0, 0.03)
	Nonglang	209	1	0.48 (0, 1.42)
	Pyndengsohstap	19	0	0 (0, 0.16)
	Siangra	66	7	10.61 (3.18, 18.04)
	Umthlu	120	4	3.33 (0.12, 6.54)
	Umwahlang	193	1	0.52 (0, 1.53)
Total		1274	17	1.33 (0.7, 1.96)

### Comparison of 2014–2017 PHC incidence with 2018 PHC and active detection estimates

Further evidence of declining incidence comes from comparing PHC-based malaria incidence for 2014–2017 with PHC results and active surveillance for 2018 (Table 3). All but two villages experienced a decline from the PHC-based incidence for 2014–2017 (average) to that for 2018. This represented a 50% or greater reduction in some villages. Only Barato E and Mukroh B villages had a slight increase in 2018, primarily reflecting small or variable numbers. Active surveillance prevalences for each village in 2018 were generally also lower than the PHC average incidence for the preceding years, except for Barato A, Kriangrin, and Siangra, which increased slightly. This may reflect chance variation because of smaller numbers of active surveillance participants.

**Table 3:** Annual village-level malaria incidence and prevalence per 100 people for nine villages of West Jaintia Hills, and 11 villages of West Khasi Hills for 2014–2018

District	Village	Malaria incidence		Prevalence
		PHC-Based 3-yr. Avg.* (2014–2017)	PHC-Based (2018)	CSCMi (2018)
West Jaintia Hills	Barato A	3.46	2.51	4.11
	Barato B	3.89	1.84	0
	Barato C	3.51	1.51	0
	Barato E	0.12	1.23	0
	Iongkwang	4.60	2.86	0
	Kynshur	1.81	1.09	0
	Mukroh A	5.67	2.80	0
	Mukroh B	3.87	5.24	0
	Saba	3.69	1.19	0
Unadjusted average		3.40	2.25	0.46
West Khasi Hills	Khylemsangrin	5.97	0.73	2.7
	Kriangrin	0.22	0.00	0.33
	Kyndongnei	2.14	0.00	0
	Langja	3.78	0.14	0
	Mawlan B	4.89	0.00	0
	Mawsikar	0.78	0.21	0
	Nonglang	23.74	10.96	0.48
	Pyndengsohstap	7.09	0.00	0
	Siangra	1.20	0.00	10.61
	Umthlu	5.61	0.25	3.33
	Umwahlang	3.80	0.18	0.52
Unadjusted average		5.38	1.13	1.63

*West Jaintia Hills-Avg. Malaria Incidence for 2014, 2015, 2017

West Khasi Hills-Avg. Malaria Incidence for 2015–2017

### *Anopheles* mosquitoes captured at participants’ houses

**Fig. 3:**
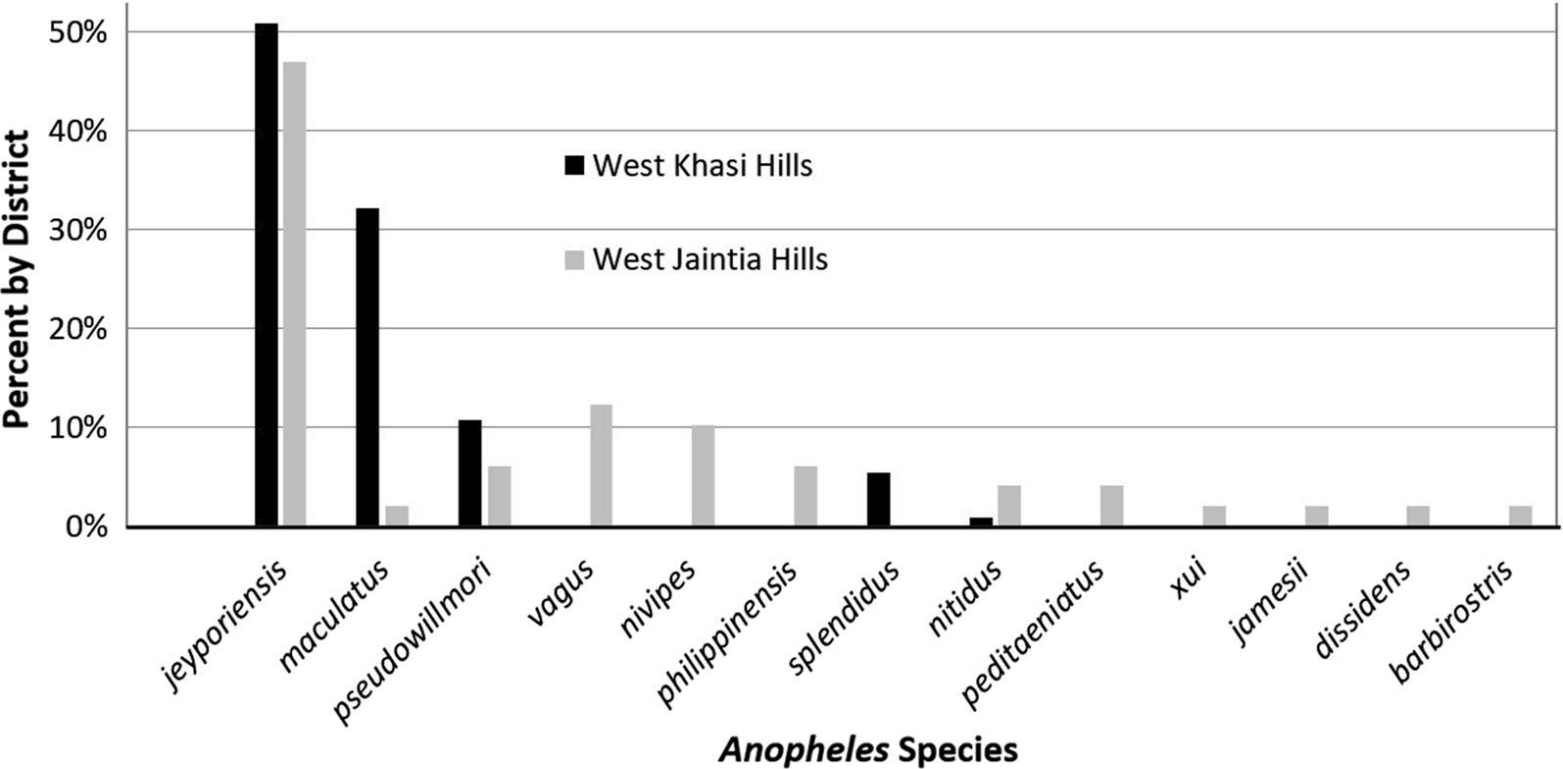
Proportion of female *Anopheles* mosquitoes by species captured in the districts of West Jaintia Hills (N = 49) and West Khasi Hills (N = 112), Meghalaya, India, during August-November, 2018

Light-trap samples from 30 houses produced 112 adult female *Anopheles* mosquitoes in West Khasi Hills (3.7 per trap-nights), where five species were identified (Table 4). In West Jaintia Hills, however, 23 houses that were sampled produced 12 species of *Anopheles* from only 49 female mosquitoes collected (2.1 per trap-nights). Interestingly, *An. jeyporiensis* was the most abundant in both West Khasi Hills (1.9 per trap-night) and West Jaintia Hills (1.0 per trap-nights), even though it has not been frequently reported in recent studies in the region. *Anopheles maculatus* and *An. pseudowillmori* were more abundant in West Khasi Hills as compared to West Jaintia Hills. Additionally, many of the species associated with rice paddies, including those of the *An. annularis*, *An. hyrcanus* and *An. barbirostris* groups, were only found in West Jaintia Hills, whereas, *An. splendidus* was captured solely in West Khasi Hills. The relative abundances of each species (Fig. 3) showed that there were few individuals for most species; however *An. jeyporiensis* was abundant in both districts, as was *An. maculatus* in West Khasi Hills. More extensive sampling will be needed to produce reliable and meaningful estimates of species diversity in the area.

**Table 4:** Species of *Anopheles* mosquitoes captured during August – November 2018 in two villages of West Khasi Hills and three villages of West Jaintia Hills

Village (Month)	West Khasi Hills			West Jaintia Hills			
	Kyrdum (Oct)	Nonglang (Nov)	Total/10 trap-nights	Barato-A (Aug)	Barato-B (Aug-Nov)	Iongkwang (Nov)	Total/10 trap-nights
(N = HHs sampled)	(N = 14)	(N = 16)	–	(N = 3)	(N = 15)	(N = 5)	–
*Anopheles species*							
*jeyporiensis*	32	25	19.0	2	15	6	10.0
*maculatus*	32	4	12.0	0	0	1	0.4
*pseudowillmori*	6	6	4.0	0	2	1	1.3
*nivipes*	0	0	0.0	0	4	1	2.2
*philippinensis*	0	0	0.0	0	1	2	1.3
*barbirostris*	0	0	0.0	0	0	1	0.4
*dissidens*	0	0	0.0	0	1	0	0.4
*nitidus*	1	0	0.3	0	0	2	0.9
*peditaeniatus*	0	0	0.0	0	2	0	0.9
*xui*	0	0	0.0	0	0	1	0.4
*vagus*	0	0	0.0	5	1	0	2.6
*splendidus*	1	5	2.0	0	0	0	0.0
*jamesii*	0		0.0	0	1	0	0.4
Total—all species	72	40	37.3	7	27	15	21.3
Total per trap-night—all species	5.1	2.5	–	2.3	1.8	3.0	–

Surprisingly, two species that have not previously been reported in India, *An. xui* and *An. dissidens,* were captured in West Jaintia Hills. *Anopheles xui*, a member of the *An. hyrcanus* group, has been described from China [13]. *Anopheles dissidens*, which is morphologically indistinguishable from *An. barbirostris*, is found elsewhere in mainland Southeast Asia [14].

### Village-level household characteristics

The household characteristics that were analysed by village represent the village-specific proportion of households with each characteristic (Table 5; Table 6). In both districts, more than half of the households in each village were composed of 4–7 occupants. In West Jaintia Hills villages (Table 5), the percentage of houses with electricity varied from 0 to 92%, while domestic water almost exclusively came from open wells/tanks (range: 97–100%), and toilet availability varied considerably. However, in West Khasi Hills, village electricity access was generally higher (20–98%), many households obtained water from an improved source, and the prevalence of household toilets was generally higher (Table 6). These same West Khasi Hills villages had a higher prevalence of houses constructed of wood/mud/thatch walls (59–100%) than those in West Jaintia Hills (37–65%).

**Table 5:** Village level household characteristics, West Jaintia Hills

Variable	Percentage of HHs per Village by Variable and Characteristic								
Characteristic	Barato A	Barato B	Barato C	Barato E	Iongkwang	Kynshur	Mukroh A	Mukroh B	Saba
Family members									
1–3	18.7	32.3	12.3	11.4	28.1	7.0	20.0	26.5	16.7
4–7	68.2	54.8	78.5	74.3	68.8	79.0	75.0	64.7	63.3
≥8	13.1	12.9	9.2	14.3	3.1	14.0	5.0	8.8	20.0
Electricity									
Yes	78.5	91.9	55.4	34.3	0	74.4	48.6	32.4	40.0
No	21.5	8.1	44.6	65.7	100	25.6	51.4	67.6	60.0
Toilet									
Yes	95.3	14.5	70.8	82.9	6.2	100	63.3	76.5	76.7
No	4.7	85.5	29.2	17.1	93.8	0	36.7	23.5	23.3
Source of water supply									
Indoor plumbing	0.9	0	1.5	0	0	0	0	0	3.3
Open well	98.1	100	96.9	100	100	100	100	100	96.7
Stream/river	0	0	1.5	0	0	0	0	0	0
Public tap	0.9	0	0	0	0	0	0	0	0
House wall material									
Concrete/brick/stone	63.5	41.9	35.4	42.8	37.5	39.5	46.6	58.8	40.0
Wood/Mud/thatched	36.5	58.1	64.6	57.2	62.5	60.5	53.4	41.2	60.0
House roof material									
Concrete/tiles	10.3	3.2	0	2.9	0	0	0	8.8	0
Tin	86.0	90.3	75.4	85.7	100	93.0	85.0	79.4	76.7
Thatched/others	3.7	6.5	24.6	11.4	0	7.0	15.0	11.8	23.3
Domestic animals present									
Yes	53.3	50.0	78.5	65.7	37.5	81.4	55.0	44.1	73.3
No	46.7	50.0	21.5	34.3	62.5	18.6	45.0	55.9	26.7
Mosquitos indoor									
Yes, always	41.1	61.3	78.5	68.6	43.8	67.4	45.0	44.1	70.0
Yes, sometimes	15.9	38.7	12.3	31.4	56.2	32.6	11.7	55.9	23.3
Only rainy season	43.0	0	9.2	0	0	0	43.3	0	6.7
Observed bed nets (N)									
1–4	64.5	77.4	70.8	68.6	100	74.4	90.0	76.5	60.0
≥ 4	35.5	22.6	29.2	31.4	0	25.6	10.0	23.5	40.0
Households with ITN									
Yes	100	100	100	100	100	100	98.3	100	100
No	0	0	0	0	0	0	1.7	0	0
IRS of HH/compound									
Yes	0.9	0	0	0	31.2	25.6	6.7	8.8	0
No	99.1	100	100	100	68.8	74.4	93.3	91.2	100
Used ≥ preventive measure									
Yes	81.3	41.9	64.6	11.4	56.2	83.7	33.3	58.8	80.0
No	18.7	58.1	35.4	88.6	43.8	16.3	66.7	41.2	20.0
Fever treated at PHC									
Public	99.1	100	100	100	100	100	98.3	100	100
Private	0.9	0	0	0	0	0	1.7	0	0

**Table 6:** Village level household characteristics, West Khasi Hills

Variable	Percentage of HHs per village by variable and characteristic											
Characteristic	Khyllemsangrin	Kriangrin	Kyndongnei	Kyrdum	Langja	Mawlan B	Mawsikar	Nonglang	Pyndengsohstap	Siangra	Umthlu	Umwahlang
Family members												
1–3	6.9	17.6	8.0	8.0	7.7	9.1	17.9	5.4	20.0	4.5	24.2	4.0
4–7	62.1	67.6	60.0	58.0	61.5	63.6	64.3	75.0	80.0	77.3	60.6	64.0
≥ 8	31.0	14.8	32.0	34.0	30.8	27.3	17.8	19.6	0	18.2	15.2	32.0
Electricity												
Yes	82.8	91.2	88.0	60.0	76.9	54.5	67.9	98.2	20.0	81.8	90.9	70.0
No	17.2	8.8	12.0	40.0	23.1	45.5	32.1	1.8	80.0	18.2	9.1	30.0
Toilet												
Yes	82.8	97.1	100	94.0	76.9	90.9	100	100	100	90.9	90.9	94.0
No	17.2	2.9	0	6.0	23.1	9.1	0	0	0	9.1	9.1	6.0
Source of water supply												
Indoor plumbing	3.4	0	8.0	16.0	7.6	27.3	0	0	0	0	6.1	0
Bore well	0	0	4.0	4.0	0	0	0	1.8	0	40.9	15.2	8.0
Open Well	10.3	0	12.0	12.0	46.2	45.5	0	7.1	100	36.4	15.2	62.0
Stream/River	0	4.7	48.0	56.0	0	9.1	0	0	0	18.2	9.1	26.0
Public Tap	86.2	85.31	28.0	12.0	46.2	18.2	100	91.1	0	4.5	54.5	4.0
House wall material												
Concrete/Brick/stone	20.6	41.2	36.0	6.0	30.8	0	21.4	19.6	0	13.6	21.2	12.0
Wood/Mud/thatched	79.4	58.8	64.0	94.0	69.2	100	78.6	80.4	100	86.4	78.8	88.0
House roof material												
Concrete/Tiles	3.4	0	0	2.0	15.4	0	0	3.6	0	4.5	6.0	0
Tin	93.2	100	100	96.0	69.2	81.8	100	96.4	100	95.5	87.9	94.0
Thatched/others	3.4	0	0	2.0	15.4	18.2	0	0	0	0	6.1	6.0
Domestic animals present												
Yes	65.5	50.0	72.0	86.0	87.5	100	75.0	44.6	100.0	31.8	39.4	44.0
No	34.5	50.0	28.0	14.0	12.5	0	25.0	55.4	0	68.2	60.6	56.0
Mosquitos indoor												
Yes, always	34.5	5.9	32.0	46.0	30.8	63.6	7.2	48.2	0	9.1	51.5	20.0
Yes, sometimes	17.2	20.6	32.0	42.0	38.4	36.4	82.1	17.9	100	9.1	9.1	22.0
Only in rainy season	48.3	73.5	36.0	12.0	30.8	0	10.7	33.9	0	81.8	39.4	58.0
Observed bed nets (N)												
1–3	51.7	79.4	64.0	62.0	58.3	72.7	92.9	75.0	100	81.8	78.1	76.0
≥ 4	48.3	20.6	36.0	38.0	41.7	27.3	7.1	25.0	0	18.2	21.9	24.0
Households with ITN												
Yes	100	100	100	98.0	75.0	100	89.3	100	100	100	100	96.0
No	0	0	0	2.0	25.0	0	10.7	0	0	0	0	4.0
IRS of HH/compound												
Yes	75.9	5.9	56.0	62.0	53.8	45.5	42.9	46.4	0	59.1	50	50
No	24.1	94.1	44.0	38.0	46.2	54.5	57.1	53.6	100	40.9	50	50
Used ≥ preventive measure												
Yes	44.8	32.4	48.0	16.0	81.2	0	25.0	55.4	60.0	63.6	66.7	24.0
No	55.2	67.6	52.0	84.0	18.8	100	75.0	44.6	40.0	36.4	33.3	76.0
Fever treated at PHC												
Public	86.2	64.7	80.0	94.0	76.9	100	100	94.6	100	50	66.7	96.0
Private	13.8	35.5	20.0	6.0	7.7	0	0	5.4	0	50	27.3	4.0
Health worker	0	0	0	0	15.4	0	0	0	0	0	6.1	0

Villages also differed in the prevalence of households with reported mosquitoes present and in their anti-mosquito practices. In West Jaintia Hills villages (Table 5), most people reported that mosquitoes were "always" present in the house (41–79%), and virtually every household (98–100%) had one or many ITNs. In addition, households in these villages more often used multiple malaria preventions, and virtually every household used a "public" health centre for fever treatment. In contrast, in West Khasi Hills villages (Table 6), a lower prevalence of houses reported that mosquitoes were "always" present (0% to 64%), and the prevalence of one or more ITNs was lower (75–100%). Fevers were more often treated at a "private" health facility.

### Village-level risk factors for *Plasmodium* infection

For statistical analyses, any village with one or more residents who tested positive by PCR for *Plasmodium* infection in the 2018 active surveillance was designated as a *Plasmodium*-positive village. Bivariate logistic regression of all 21 villages comparing village-level infection (Y/N) with village-level prevalence of each covariate, here defined as percentage of households in the village reporting the covariate, showed significant positive associations of *Plasmodium* infection with presence of mosquitoes, presence of electricity, and absence of domestic animals (Table 7). A multivariate logistic regression model that controlled for various possible confounders did not result in any significant associations.

**Table 7:** Logistic regression of village-level risk factors for *Plasmodium falciparum* infection for 21 villages in West Khasi and West Jaintia Hills, Meghalaya, 2018

Village characteristics (% of households)	Crude odds ratio (95% CI)	*p*-value
Presence of electricity	1.11 (1.01, 1.23)	0.04
Presence of toilet	1.07 (0.97, 1.19)	0.18
Housing material: Wood	1.01 (0.96, 1.07)	0.64
Roofing material: Thatched	0.84 (0.69, 1.03)	0.93
Presence of animals	0.91 (0.84, 0.99)	0.03
Mosquitoes	1.19 (1.12, 1.39)	0.03
IRS sprayed	0.16 (0.99, 1.07)	0.16
Net used by 1–3 people	0.98 (0.91, 1.05)	0.51
Use of preventive measures	1.01 (0.97, 1.05)	0.62
Animals kept inside the house	0.95 (0.87, 1.01)	0.10

### Spatial distribution of village-level prevalence of *Plasmodium* infected participants indicates spatial clustering in the West Khasi Hills

**Fig. 4:**
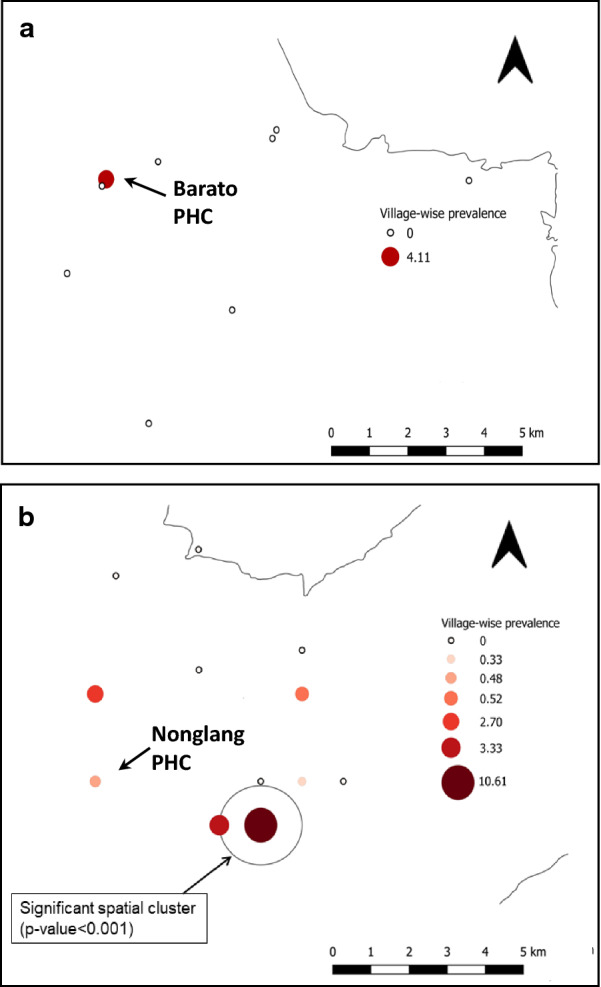
Spatial distribution of *Plasmodium* infection prevalence in study villages surrounding **a** Barato PHC, West Jaintia Hills and **b** Nonglang PHC, West Khasi Hills

The geographic locations of each village, and the proportion of residents with *P. falciparum* infection (circle size) are shown in Fig. 4. In the West Jaintia Hills (Fig. 4a), only one village (Barato A) had any infections. In the West Khasi Hills (Fig. 4b), village-level infection prevalence ranged from 0 to 10.6%. Significant clustering of infection (observed = 11, expected = 2.15, Relative Risk (RR) = 12.65; p < 0.001) with a radius of 1 km was observed in southeastern part of the catchment area (Fig. 4b).

## Discussion

This study investigated the spatial and temporal prevalence of *Plasmodium* infections among villages in two districts of Meghalaya state in northeast India in the context of recently declining malaria incidence. This is the first active surveillance-based report on malaria in the region using PCR-based methods to determine infection status. These community surveys occurred after a widespread distribution of LLINs throughout the region in 2016 that is believed to have contributed to declining malaria incidence recorded through PHC-based passive surveillance. Even with declining incidence, inter-annual variation among villages in *Plasmodium* infection prevalence was observed, along with risk factors that partly explain the patterns identified.

PHC data for the period 2014–2018 showed a general decline in the number of malaria cases, but this varied among villages. These results expand on the recent district-level decline described by Kessler et al*.* [4] by showing how some districts experienced little or no waning while others dropped quickly. Village-level information on the government's LLIN programme indicates that LLINs were distributed in the West Jaintia Hills and West Khasi Hills survey villages during April 2016 and May 2016, respectively. The NVBDCP in Meghalaya reported that wide-scale distribution took place throughout 2016 in all districts of the state. Hence, the general decline in PHC-based incidence may be linked to increased use of LLINs, either alone or in combination with other prevention and control measures such as indoor residual spray (IRS). Studies conducted in India [15] have demonstrated that use of LLINs and IRS [16] results in reduced malaria incidence. In India, LLINs have been evaluated for duration of effectiveness and acceptability, with very promising results. LLINs distributed in malaria-endemic communities of neighbouring state Assam during 2009–2013, for example, were found to be effective for up to three years, and community members recognized the protection benefits of these nets and regularly used them [17].

Another study during 2009 in Uttar Pradesh indicated that people considered LLINs to be safe, effective, and socially acceptable [18]. Investigations in central India (Chhattisgarh) during 2006–2007 demonstrated that Interceptor LLINs were highly effective in reducing *An. culicifacies* densities and decreasing human malaria incidence [19]. Although LLINs are considered an effective malaria prevention tool in India, more detailed analyses of household- and individual-level level risk factors are needed to determine whether particular conditions or behaviours might help explain infection patterns that were observed among villages in this study [20, 21].

Malaria diagnosis at the study PHCs, and throughout Meghalaya, is mainly performed using slide microscopy and RDTs. Due to declining *Plasmodium* transmission, microscopy and RDTs may not be sensitive enough to detect all cases of parasitaemia [22] resulting in underestimation of true prevalence, particularly if infection is increasingly subpatent and asymptomatic [23]. PCR detects more than twice the number of *Plasmodium* infections compared to microscopy and RDTs [24] and is very effective at detecting low-density infections [25]. In this study, current infection was determined by PCR to improve detection of low-level parasitaemia and/or asymptomatic infections. As the number of new infections in the study region continue to decline, PCR represents a more sensitive test [26] to better identify unrecognized, potentially infectious, carriers who can be treated, thereby reducing continued *Plasmodium* transmission. Indeed, elimination efforts in India will depend on improved diagnosis and treatment of asymptomatic infections combined with enhanced vector control and more focused disease surveillance [23, 27].

As a component of surveillance, analysis of spatial patterns can serve many purposes, including data exploration, visualizing configurations, defining spatial resolution, determining clusters, developing causal hypotheses, and more (e.g., [28]). In this study, a simple spatial statistical analysis was undertaken to evaluate village-level geographic clustering of *Plasmodium* infection prevalence. Similar spatial clustering of malaria village-level prevalence has been used in other regions, such as Ethiopia [29], Solomon Islands [30], and Bangladesh [31], but rarely in India [32]. Here, a small cluster of two villages was found in the southeastern part of West Khasi Hills. Otherwise, village-level prevalence was generally low and heterogeneously distributed. Nevertheless, village-level surveillance should play an important role in monitoring transmission as regional elimination possibilities increase [27].

Local environmental risk factors such as proximity to water bodies, elevation, topography, and land use/land cover have been shown to explain spatial clustering of higher prevalence locations [33, 34]. The results presented here suggest that the use of spatial tools and satellite-derived environmental data might help policy makers to formulate targeted intervention strategies for future malaria control and elimination. More generally, where higher-incidence villages are clustered, anti-malarial interventions such as ITNs and IRS can be targeted to those persistent hot spots, eliminating wasteful spending [30, 35]. However, interventions that reduce vector abundance and infection incidence will increase the challenge of identifying foci of transmission as incidence declines [30].

Villages with more households that kept domestic animals at the house had lower odds of *Plasmodium* infection, which is similar to findings in a study conducted in Zambia [36]. This finding, however, is in contrast to other research conducted in Indonesia [37] that suggested keeping livestock in the house increased malaria risk. Domestic animals may attract zoophilic mosquitoes away from humans, reducing people's exposure to infectious bites [38]. However, the availability of non-human blood meals often increases vector density and longevity [39], possibly enhancing malaria risk. Similarly, the presence of livestock animals nearby houses may increase the olfactory signal that attracts host-seeking female vectors, thereby increasing the risk of humans being bitten by infected mosquitoes. From the analyses, it is unclear why greater village-level presence of animals in the households studied here appeared to be protective, and more detailed analyses at the household level will be instructive. Where zoophilic vectors are abundant, existing vector control measures may be inadequate as part of efforts aimed at malaria elimination, and targeting livestock structures such as cattle sheds may increase the potential for malaria elimination [40].

Village-level *Plasmodium* infection prevalence was higher in villages with a greater proportion of households that had access to electricity. In some developing country settings, electricity availability is an indicator of higher SES, which is often related to lower risk of malaria. Most studies recognize malaria as a disease of poverty, as the malaria burden is often concentrated in the poorest continents and countries. In the study area, perhaps presence of electricity in these rural households attracts *Anopheles* vectors [41], thereby increasing the likelihood of transmission. Interestingly, a study conducted in Burkina Faso [42] showed that households with access to electricity were more likely to experience malaria. More detailed, household-level analyses are needed to better understand how presence of electricity might alter malaria risk.

Many other studies have revealed how different types of housing material used in the construction of roofs and walls are associated with malaria incidence [42]. However, in this study, no association between roof and wall materials was found which is consistent with the results from other studies [23]. In contrast, investigations in Assam, India [43] demonstrated that malaria incidence was higher among people residing in bamboo houses. The lack of association of housing material in this study may be attributable to little variation across these variables. Alternatively, house construction materials may be less important if biting often occurs outside or if LLINs are widely and properly used. More generally, focused studies are needed on specific features of housing and socio-economic development that reduce malaria risk in different contexts.

Studies conducted in India [20] have shown that the lack of mosquito prevention methods at the village level, such as use of ITNs or IRS, are risk factors for malaria. Curiously, the village-level prevalence of mosquito prevention efforts (e.g., coils, ITNs or IRS) was not associated with *Plasmodium* infection prevalence. Perhaps there was insufficient variation among villages in use of these prevention measures [44] or possibly inadequate or improper application. Finer-scale analyses at the household level (e.g., [44]) are needed to further investigate the impacts of these interventions [30].

A total of 13 *Anopheles* species were captured despite limited sampling. This is consistent with previous studies in the region that have identified a high diversity of anophelines, some of which were involved in malaria transmission [13, 45–48]. These potential vectors, many of which are broadly zoo-anthropophilic, are found in diverse habitats and specifically forest-fringe and rice-growing environments. *Anopheles* species diversity was greater in the West Jaintia Hills than in the West Khasi Hills. This may be because the former is relatively warmer and at generally lower elevation, but further studies in other villages and seasons are needed to determine if this is a general pattern. The predominance of *An. jeyporiensis*, which has not often been reported in the region in recent years, is noteworthy.

Two species historically considered as primary vectors in the region, *An. baimaii* and *An. minimus,* were not identified in the samples, which corroborates more extensive recent studies in northeast India that these forest-associated mosquito species are in decline [47, 48]. There is growing concern that species previously considered to be secondary vectors may be playing an increasingly important role in malaria transmission though this remains to be characterised [3]. In this respect, *An. jeyporiensis* and *An. maculatus* are of particular concern as they were the most abundant species in this study and both have been reported to be infected with *P. falciparum* in this region*,* specifically: *An. jeyporiensis* in Assam [49] and Bangladesh [45] and in studies from 80 years ago, *An. maculatus* from Assam and Meghalaya [50, 51]. In the context of changing malaria epidemiology due to deforestation and increased LLIN use, further studies are needed to determine which species are now contributing to malaria transmission and to characterize their biological attributes relevant to vector control including biting-time, host-preference, larval ecology, and seasonal abundance.

Although this study offers important findings, it also has some limitations. First, it was not possible to evaluate all possible risk factors that may contribute to the geographic variation in *Plasmodium* infection risk and the presence of disease clustering, such as *Anopheles* mosquito abundance and distribution, distances from houses to water bodies or rice paddies, or elevation. Second, no data on weather variables such as temperature, precipitation, and/or humidity were included, which could alter vector-human contact and impact malaria parasite prevalence [52]. Finally, although this study focused on two districts of Meghalaya, it is difficult to generalize findings to the entire state or other parts of north-east India [3].

## Conclusion

This study demonstrated that malaria incidence has recently declined perhaps in part due to the widespread introduction of control strategies, especially LLINs. Nevertheless, temporal and spatial variation has left some villages at higher risk, making efforts aimed at regional elimination more difficult. Therefore, anti-malarial interventions should be continued, even expanded, if the government goal of malaria elimination by 2030 is to be achieved. The second round of state-wide LLIN distribution in Meghalaya was scheduled for 2020 but paused due to the COVID-19 pandemic. Malaria-prevention education for people of all ages in these communities should be pursued through different educational mediums. Because of cross-border movement of people and parasites [53, 54], the goal of malaria elimination in India must be a multinational, regional effort [2, 55]. More detailed risk factor analysis at the household and individual levels, combined with expanded environmental and spatial analyses, should help to detect high risk settings and "silent" transmission areas, thereby assisting the NVBDCP and government policy makers to plan, design, and streamline malaria preventive measures.

## Data Availability

Data generated in this study will be made available through the open-access online resource for population-based epidemiological studies ClinEpiDB (https://clinepidb.org). Meghalaya State Malaria Control Programme provided the state malaria surveillance data.

## Abbreviations

API: Annual parasite index
ASHA: Accredited social health activist
ITNs: Insecticide-treated nets
IRS: Indoor residual spray
LLINs: Long-lasting insecticide-treated bed nets
NVBDCP: National Vector Borne Disease Control Programme
PCR: Polymerase chain reaction
PHC: Primary health centre
